# Distinguishing benign and malignant myxoid soft tissue tumors: Performance of radiomics vs. radiologists

**DOI:** 10.1371/journal.pone.0318072

**Published:** 2025-01-27

**Authors:** Joshua M. Lawrenz, Can Cui, Samuel R. Johnson, Katherine S. Hajdu, Stephen W. Chenard, Akhil Rekulapelli, Cullen P. Moran, John J. Block, Nicholson S. Chadwick, Joanna L. Shechtel, Brian Bingham, Leo Y. Luo, Jennifer L. Halpern, Herbert S. Schwartz, Ginger E. Holt, David S. Smith, Benoit Dawant, Hakmook Kang

**Affiliations:** 1 Department of Orthopaedic Surgery, Division of Musculoskeletal Oncology, Vanderbilt University Medical Center, Nashville, Tennessee, United States of America; 2 Vanderbilt Institute of Surgery and Engineering, Vanderbilt University Medical Center, Nashville, Tennessee, United States of America; 3 Department of Radiology, Division of Musculoskeletal Radiology, Vanderbilt University Medical Center, Nashville, Tennessee, United States of America; 4 Department of Radiation Oncology, Vanderbilt University Medical Center, Nashville, Tennessee, United States of America; 5 Department of Biostatistics, Vanderbilt University Medical Center, Nashville, Tennessee, United States of America; IBM Research - Israel, ISRAEL

## Abstract

**Introduction:**

Benign and malignant myxoid soft tissue tumors have shared clinical, imaging, and histologic features that can make diagnosis challenging. The purpose of this study is comparison of the diagnostic performance of a radiomic based machine learning (ML) model to musculoskeletal radiologists.

**Methods:**

Manual segmentation of 90 myxoid soft tissue tumors (45 myxomas and 45 myxofibrosarcomas) was performed on axial T1, and T2FS or STIR magnetic resonance imaging sequences. Eighty-seven radiomic features from each modality were extracted. Five ML models were trained to classify tumors as benign or malignant in 40 tumors and then tested with an additional 50 tumors using cross validation. The accuracy of the best ML model based on area under the receiver operating characteristic curve (AUC) was compared to the consensus diagnosis of three musculoskeletal radiologists. Correlation between radiologist confidence (equivocal, probably, consistent with) and accuracy was tested.

**Results:**

The best ML classifier was a logistic regression model (AUC 0.792). Using T1 + T2/STIR images, the ML model classified 78% (39/50) of tumors correctly at a similar rate compared to 74% (37/50) by radiologists. When radiologists disagreed, the consensus diagnosis classified 50% of tumors (7/14) correctly compared to 86% (12/14) by the ML model, though this did not reach statistical significance. Radiologists had a cumulative accuracy of 91% (30/33) when they rated their confidence ‘consistent with’ compared to 61% (31/51) when they rated their confidence ‘equivocal/probably’ (P = 0.006). For cases when radiologists rated their confidence ‘equivocal/probably’, the ML model had 76% accuracy (39/51).

**Conclusions:**

A radiomic based ML model predicted benign or malignant diagnosis in myxoid soft tissue tumors similarly to the consensus diagnosis by three musculoskeletal radiologists. Radiologist confidence in the diagnosis strongly correlated with their diagnostic accuracy. Though radiomics and radiologists perform similarly overall, radiomics may provide novel diagnostic utility when radiologist confidence is low, or when radiologists disagree.

## Introduction

Distinguishing benign and malignant myxoid soft tissue masses in the musculoskeletal system can be challenging due to the shared clinical, imaging, and histologic features [1]. Magnetic resonance imaging (MRI) often shows a classic appearance of low T1 signal and very high homogeneous T2 signal secondary to their high-water content. There also exists histologic overlap that lends itself to the potential for tissue sampling error with core needle biopsy, particularly with myxomas and low grade myxofibrosarcomas [2]. GNAS1 can be helpful to distinguish myxoma from low grade myxofibrosarcoma, though only in 50% of cases [2]. Prior studies have attempted to identify conventional MRI features to qualitatively distinguish malignant from benign tumors, such as ill-defined tumor margins or the tail sign, though the import of this imaging finding may be limited by the rather subjective assessment of human interpretation [3,4]. Further, Klein et al. in 2021 reported a 27% (5/19) rate of indeterminate diagnosis from needle biopsies in myxofibrosarcomas compared to a 13% (19/196) rate of indeterminate diagnosis from needle biopsies in a sample of 196 other soft tissue sarcomas [5]. For these reasons, even experienced multidisciplinary teams of clinicians may face uncertainty in the diagnosis of myxoid soft tissue tumors.

Radiomics is a method of machine learning (ML) that consists of large data extraction from medical images, including their qualitative and quantitative features which can be utilized for predictive or analytical purposes [6,7]. There have been few studies to date assessing the performance of MRI radiomics in diagnosing extremity soft tissue sarcomas [8–12] with two emphasizing myxoid tumors [8,10]. Key limitations of these studies were either: the use of T1 weighted images only [10], neglecting the T2 hyperintense perilesional edema or “tail-sign” that has been described in myxofibrosarcomas [3]; no performance comparison to a consensus diagnosis by radiologists [8,10]; the lack of clinical features within their predictive models [8,10]; or, the use of only two-dimensional radiomic analysis [8]. To mitigate these limitations, we previously developed five ML classifiers or models based in three-dimensional MRI radiomics from T1 and T2 weighted images in a pilot cohort of 40 myxoid tumors [13]. The highest AUC achieved was 0.780, and all models with radiomic and clinical features outperformed the classification models using clinical features alone. The rationale for the present study is to validate these preliminary findings in a separate cohort of tumors and compare the diagnostic performance of a radiomic based ML model to the consensus diagnosis of fellowship-trained musculoskeletal radiologists.

Thus, with this study we aim to answer two questions: (1) How does a radiomic based ML model’s performance compare to the consensus diagnosis of three musculoskeletal radiologists? (2) How confident are radiologists in the diagnosis and does their confidence level correlate with accuracy?

## Methods

### Patient cohort and study design

**Fig 1:**
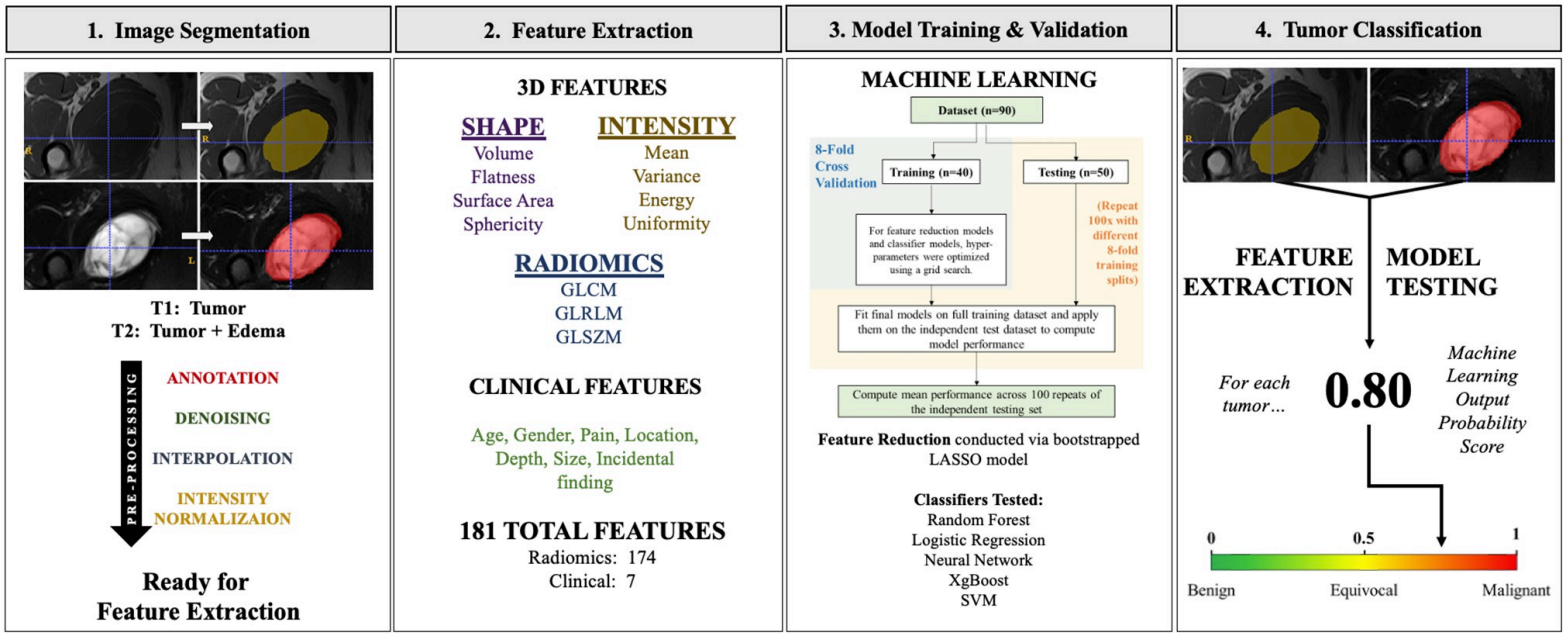
Flowchart of radiomic-based machine learning model development. In step 1, MR images are manually segmented by attending clinicians. In step 2, feature extraction is performed using a LASSO model. In step 3, classifiers are trained and tested using cross-validation. In step 4, a probability score ranging from 0–1 is produced for each tumor (0 = benign; 1 = malignant).

This study received institutional review board exemption from the Human Research Protections Program at Vanderbilt University Medical Center, Nashville, TN, USA (IRB# 201841), as this study posed minimal risk to participants and thus informed consent was waived. We identified 442 musculoskeletal myxoid soft tissue tumors in our orthopaedic oncology clinical database between 1995–2020, in which 439 had surgical resection or re-resection surgery at our institution and a histologically confirmed myxoid soft tissue tumor on resection pathology. Of these, 264 were either myxomas or myxofibrosarcomas, and 165 had a pre-treatment MRI available in our imaging system. Due to incomplete or poor-quality images, 33 patients were excluded. Of the remaining 132 patients, a retrospective review of 90 patients (45 myxomas and 45 myxofibrosarcomas) who had complete imaging and clinical data was performed between June 1, 2020, and March 1, 2022. The authors had access to patient health information to obtain clinical information from the electronic medical record. Seven baseline clinical features were collected from the medical record including patient age, sex, tumor size (cm, greatest dimension), tumor depth (superficial to fascia, or deep to fascia), tumor location (upper extremity, lower extremity, or trunk), pain at presentation (yes or no), and tumor as an incidental finding on presentation (yes or no). The study design consisted of the following stages including manual tumor segmentation, radiomic feature extraction, model training and validation, and performance comparison to radiologist MRI interpretation. Clinical characteristics of the patient cohort are shown in Table 1, and the study design is shown in Fig 1.

**Table 1:** Demographic and clinical features of the benign and malignant groups.

	Benign n = 45 (%)	Malignant n = 45 (%)	P-value
Age, yrs	56.9 (20.6)	61.6 (17.3)	**0.029**
Sex			**0.008**
Male	9 (20)	22 (49)	
Female	36 (80)	23 (51)	
Size, cm	5.4 (4.4)	6.4 (5.1)	0.124
Depth			0.054
Deep to fascia	43 (96)	36 (80)	
Superficial to fascia	2 (4)	9 (20)	
Location			0.286
Upper extremity	6 (13)	8 (18)	
Lower extremity	38 (84)	33 (73)	
Trunk	1 (3)	4 (9)	
Pain			0.286
Yes	22 (49)	16 (36)	
No	23 (51)	29 (64)	
Incidental Finding			0.230
Yes	9 (20)	4 (9)	
No	36 (80)	41 (91)	

Bolded P-values are significant with P < 0.05. For continuous variables, median and IQR (interquartile range) are reported.

### MRI sequences, segmentation and normalization

All patients included in this analysis had both T1 sequences, and either T2 fat saturation (T2FS) or STIR sequences. Pre-treatment MRIs included those from our institution and outside institutions, thus sequences and slice thickness varied. Slice thickness ranged from 3.5–13.0 mm, and resolution ranged from 0.21–1.64 mm. 3D-Slicer Software (version 4.11) was used for manual segmentation of images. Manual segmentation of the entire axial T1 and entire T2FS or STIR sequences for each tumor was performed by attending clinicians (J.M.L., N.S.C., L.Y.L., J.L.S.). All 90 segmentations were reviewed and edited as necessary by a single attending musculoskeletal radiologist (J.L.S.). When performing segmentations, clinicians were blinded to clinical information and the histologic diagnosis. T1 and T2FS or STIR images were segmented separately to reduce bias of one image sequence influencing the segmentation of the other image sequence. The tumor alone was segmented on the T1 image, and the tumor plus surrounding edema was segmented on the T2FS or STIR image. In the preprocessing of each image, extreme intensity values beyond the 0.05th to the 99.95th percentile were identified as noise or outliers and subsequently clipped. The images were then resampled to a spatial resolution of 1 x 1 x 1 mm^3^, and the image intensity was normalized to a range of 0 to 255.

### Radiomic feature extraction

Radiomic features were extracted from T1 and T2FS or STIR images using PyRadiomics (version 3.0). Shape and intensity were extracted from tumor segmentations, and texture or gray level matrix features were extracted from the normalized and discretized (binwidth = 5) intensity map. Except for the seven clinical features, 87 radiomics features (shape, intensity, and texture) were extracted from each MR modality (174 features total). Z-scoring was applied to normalize these features. A least absolute shrinkage and selection operator (LASSO) model was used for feature reduction, and 80% bootstrapped samples in the training set of 40 patients were used to fit the LASSO model and repeated 100 times. For each feature, the stability score was computed in accordance with prior methods [14–16]. Then, an 8-fold cross-validation was utilized to fine-tune the threshold of the stability score for feature selection. The threshold was systematically selected from a range spanning 0 to 0.7, with increments of 0.05. Features that exceeded this threshold were subsequently selected to train the classifiers such as random forest, logistic regression, and other models. These models were then evaluated using an independent testing set of 50 patients.

**Fig 2:**
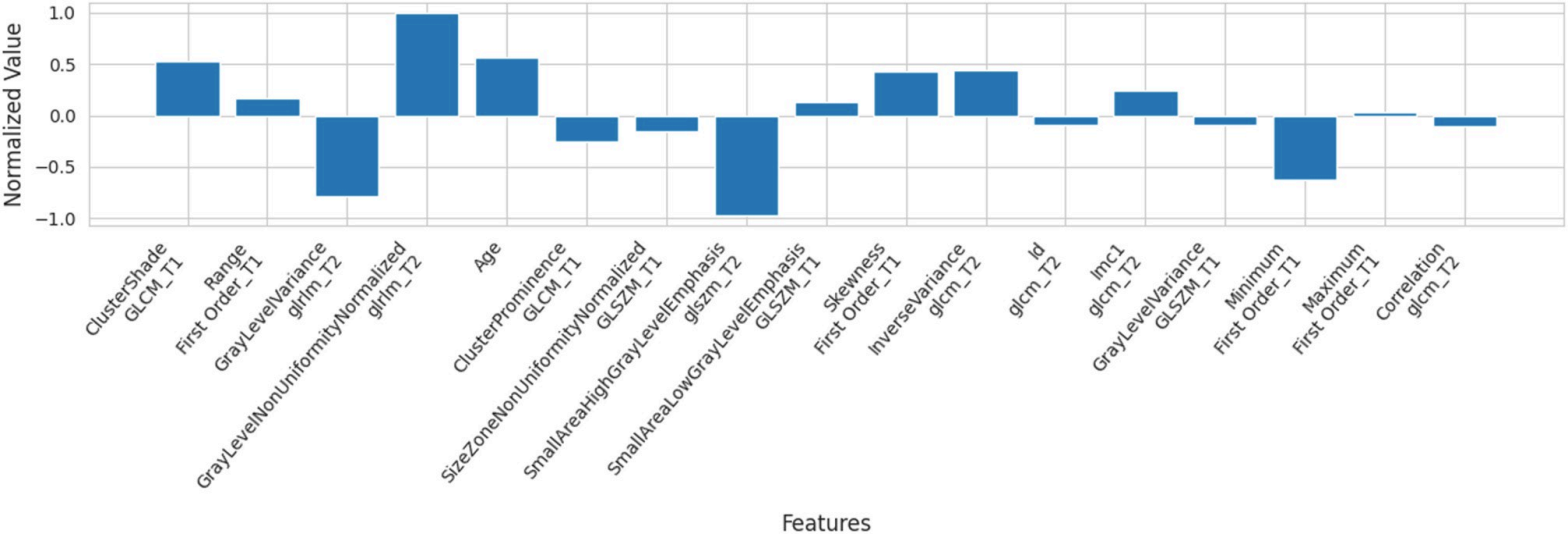
Feature importance generated by a logistic regression classifier to differentiate benign and malignant myxoid tumors. The classifier was fitted using the top 17 radiomic and clinical features, which were selected most frequently by the LASSO models. Features are arranged from left to right in decreasing order of selection frequency. The top 6 features (on the left) were selected by all LASSO models. Normalized value is the log odds ratio of each feature’s association with malignancy, i.e., the greater the magnitude of a positive value, the more likely malignant.

To further analyze feature relevance to classifiers, we calculate the normalized coefficients of the most frequently selected features. Specifically, after repeating the above feature selection and classifier training 100 times, a list of features was sorted in descending order based on their selection frequency across the 100 times. We focused on the top 17 features that were selected by more than 25 of the 100 times of feature reduction. These 17 features from the training and testing set were then employed to retrain a logistic regression classifier. The normalized coefficients of this logistic regression classifier indicate the relevance of each feature in the performance classifier, and they are shown in Fig 2.

### Model construction and validation

Model construction and validation was performed using Sklearn (version 0.22.2). With the selected radiomic and clinical features, five ML classifiers or models (random forest, logistic regression, neural network, XgBoost, and SVM) were trained to classify tumors as benign or malignant in an initial 40 patients, and then tested with a separate testing cohort of 50 patients.

Within the training set, an 8-fold cross-validation was used for parameter tuning. After tuning and training, the trained model was evaluated on an independent testing set. This process was repeated 100 times to obtain an average performance metric on the independent testing set. The information of classifier structures and parameters for tuning are listed in the S1 Appendix.

Each classifier model was constructed with reduced radiomic and clinical data. We used the predict() in scikit-learn (https://scikit-learn.org/stable/) to obtain the binary classification of benign or malignant tumors, and pred_proba() to get the prediction probability. The probability scores from pred_proba() ranging from 0 to 0.5 were classified as benign in predict(), while scores ranging from 0.5 to 1 were classified as malignant. The model was accurate when the benign or malignant classification based on the probability score was concordant with the histologic diagnosis of the tumor on final resection. Specially, for SVM classifier, the original output for class prediction returns the signed distance to the hyperplane, which is not probability. However, by setting the probability parameter of the SVM function in scikit-learn to True, we enabled the calculation of probability scores using predict_proba() (https://scikit-learn.org/stable/modules/svm.html). This approach, which includes an internal cross-validation step during training, allowed us to obtain probability estimates for the classifier. The probability outputs were used to calculate the area under the receiver operating characteristic curve (AUC) in this work, while other evaluation metrics including accuracy, sensitivity, specificity, positive predictive value (PPV), negative predictive value (NPV) were calculated using the binary classification.

### Radiologist imaging review

The same seven clinical features inputted in the ML model were provided to the radiologists when they classified each tumor. The radiologists were blinded to the final resection histologic diagnosis. Two attending musculoskeletal radiologists 1 and 2 (N.S.C., and J.L.S.) independently classified the 50 myxoid tumors in the validation cohort as benign or malignant based on the MR imaging; for discordant cases when radiologists 1 and 2 disagreed, a third senior musculoskeletal radiologist (J.J.B.) with 15 years of clinical experience made the consensus diagnosis. Radiologists classified tumors as benign or malignant with three iterations of information provided to them: 1. T1 + T2FS/STIR images; 2. T1 + T2FS/STIR + post-contrast images; and 3. T1 + T2FS/STIR + post-contrast images + the probability-based ML output score generated from the ML model. The probability-based ML output score ranged from 0 to 1 (0 = benign, 1 = malignant). The radiologists completed classifications of a tumor with all iterations of data prior to moving onto the next case and were blinded to ML performance. The radiologists were accurate when their consensus benign or malignant classification was concordant with the histologic diagnosis of the tumor. Radiologists 1 and 2 also individually rated their confidence in the diagnosis for each tumor with each imaging iteration on a three-level scale: equivocal (low confidence), probably (intermediate confidence), or consistent with (high confidence).

### Statistical analysis

Quantitative and qualitative radiomic features were compared between benign and malignant tumors using Wilcoxon-Rank sum tests and chi-squared tests, respectively. The area under the receiver operating characteristics curves (AUC) for each radiomic ML model were compared. Accuracy, sensitivity, specificity, positive predictive value (PPV), negative predictive value (NPV) was compared between the best radiomic model and the consensus diagnosis of the radiologists using a two-tailed McNemar’s test. Classification agreement between radiologist 1 and 2 for both confidence and accuracy was measured using Kappa statistics. Logistic regression was used to assess the correlation between confidence and accuracy for radiologist 1 and 2 for each of the three iterations of classification (T1+T2/STIR; T1+T2/STIR + post-contrast; and T1+T2/STIR + post-contrast + ML). All tests were two-sided, and a P-value less than 0.05 was considered statistically significant. Whenever necessary, we controlled the false discovery rate (FDR) at 0.05 to account for multiple comparisons. All statistical calculations were performed using R (version 4.0.2).

### Patient characteristics

There were 90 patients in the entire cohort, in which 45 had benign myxomas and 45 had malignant myxofibrosarcomas. The malignant cohort was slightly older, at a mean age of around 62 years compared to 57 years in the benign cohort (P = 0.029), and had relatively fewer females (51% vs. 80%, P = 0.008). As noted in Table 1, there were no other statistically significant differences in the baseline patient characteristics (tumor size, tumor depth, tumor location, pain, and tumor as an incidental finding) between the benign and malignant cohorts. The median tumor size was around 6 cm, and 88% (79/90) of tumors were deep to fascia. Most tumors were in the lower extremity (79%, 71/90). Tumors were painful in 42% (38/90) of patients and presented as an incidental finding in 14% (13/90) of patients.

## Results

### Radiomics vs. Radiologists performance

The best ML model when classifying the validation cohort of tumors was a logistic regression model with an AUC of 0.792 (0.010) (Table 2). The radiomic and clinical features most frequently selected by the logistic regression classifier are shown in Fig 2. The accuracy of the logistic regression model was 78%, while the accuracy of the radiologist consensus diagnosis was 74% (P = 0.789). The accuracy, sensitivity, specificity, PPV and NPV of radiomics, radiologist 1, radiologist 2 and the consensus radiologist diagnosis are shown in Table 3. The classification performance of radiomics was better than or equal to the consensus radiologist diagnosis in all performance metrics. There was also no difference in accuracy between radiomics and radiologist 1 (P = 0.146), radiomics and radiologist 2 (P = 0.606), and radiologist 1 and 2 (P = 0.789). When assessing the cumulative accuracy of the radiologists, radiologists 1 and 2 performed marginally worse when given post-contrast images (73%, 61/84), and marginally better when given the ML output score (76%, 64/84). When radiologists 1 and 2 were discordant (14/50), the senior radiologist classified 50% of tumors (7/14) correctly compared to 86% (12/14) by radiomics (P = 0.074). There was fair agreement (K = 0.2–0.4) between radiologist 1 and 2 in accuracy for all three iterations of classification. For classification using T1+T2/STIR images, K = 0.27 (FDR-adjusted P = 0.052); for classification using T1+T2/STIR + post-contrast images, K = 0.35 (FDR-adjusted P = 0.039); and for classification using T1+T2/STIR + post-contrast + ML images, K = 0.310 (FDR-adjusted P = 0.039).

**Table 2:** Classification performance of five ML models using T1+T2/STIR images.

	AUC (STD)
**Logistic regression**	0.792 (0.010)
**SVM**	0.792 (0.030)
**Neural network**	0.784 (0.027)
**Xgboost**	0.747 (0.032)
**Random forest**	0.739 (0.023)

AUC = area under the receiver operating characteristic curve.

**Table 3:** Classification performance of radiomics vs. radiologists in distinguishing benign and malignant myxoid tumors using T1+T2/STIR images.

**Performance**				
	**Radiomics (%)** *	**Consensus (%)**	**Radiologist 1 (%)**	**Radiologist 2 (%)**
**Accuracy**	78.0	74.0	72.0	76.0
**Sensitivity**	72.0	72.0	80.0	64.0
**Specificity**	84.0	76.0	64.0	88.0
**PPV**	81.8	75.0	69.0	84.2
**NPV**	75.0	73.1	76.2	71.0
Radiomics* **vs. Radiologists**				
	**Accuracy Comparison** ^a^		**FDR-adjusted P-Value**	
	Radiomics vs. Consensus Radiology		0.789	
	Radiomics vs. Radiologist 1		0.584	
	Radiomics vs. Radiologist 2		0.789	
	Radiologist 1 vs. Radiologist 2		0.789	

* = logistic regression radiomic model;

PPV = positive predictive value; NPV = negative predictive value;

^a^ = using McNemar test.

### Radiologist confidence

For radiologist 1 and 2, the frequency of rating of their confidence level as ‘consistent with’ increased from 26% (26/100) when using T1+T2/STIR images, to 39% (33/84) when also given post-contrast images, and to 42% (35/84) when also given ML output score (P = 0.002). Table 4 summarizes the radiologist accuracy results based on their confidence level. Using T1+T2/STIR images, radiologists 1 and 2 had a cumulative accuracy of 96% (25/26) when they rated their confidence as ‘consistent with’, compared to a cumulative accuracy of 66% (49/74) when they rated their confidence as ‘equivocal/probably’ (FDR-adjusted P = 0.006). Using T1+T2/STIR + postcontrast images, radiologists 1 and 2 had a cumulative accuracy of 91% (30/33) when they rated their confidence as ‘consistent with’ compared to cumulative accuracy of 61% (31/51) when they rated their confidence as ‘equivocal/probably’ (FDR-adjusted P = 0.006). Radiomics was found to be 76% accurate (39/51) in the cohort of patients in which the radiologists rated their confidence as ‘equivocal/probably’ (P = 0.135). Using T1+T2/STIR + post-contrast + ML output, radiologists 1 and 2 had a cumulative accuracy of 94% (33/35) when they rated their confidence as ‘consistent with’ compared to cumulative accuracy of 63% (31/49) when they rated their confidence as ‘equivocal/probably’ (FDR-adjusted P = 0.006). There was fair agreement (K = 0.2–0.4) between radiologist 1 and 2 in confidence for all three iterations of classification. For classification using T1+T2/STIR images, K = 0.28 (FDR-adjusted P = 0.006); for classification using T1+T2/STIR + post-contrast images, K = 0.23 (FDR-adjusted P = 0.037); and for classification using T1+T2/STIR + post-contrast + ML output, K = 0.34 (FDR-adjusted P < 0.001).

**Table 4:** Cumulative accuracy results based on confidence level of radiologist 1 and 2 with three iterations of diagnostic classification.

	Confidence level *‘Consistent with’*	Confidence level *‘Equivocal/probably’*	FDR-adjusted P-Value
T1+T2/STIR	96% accurate (25/26)	66% accurate (49/74)	**0.006**
T1+T2/STIR + post-contrastk*	91% accurate (30/33)	61% accurate (31/51)	**0.006**
T1+T2/STIR + post-contrast + ML output*	94% accurate (33/35)	63% accurate (31/49)	**0.006**

*Total cumulative cases viewed between radiologist 1 and 2 equals 84 instead of 100, as only 42 of 50 patients had postcontrast images available.

Bolded P-values are significant with FDR-adjusted P < 0.05.

## Discussion

In this study, we report a radiomic based ML model performed similarly to the consensus diagnosis of three musculoskeletal radiologists in the classification of benign and malignant myxoid soft tissue tumors. Radiologist confidence in the diagnosis strongly correlated with their diagnostic accuracy. In cases when radiologist confidence is low or radiologists disagree, the ML model may have utility to augment human interpretation of images, though these trends did not reach statistical significance.

To our knowledge, the present study is the first in the literature to report a radiomic based ML model constructed from both clinical features and features from T1 and T2 images, with comparison to the consensus diagnosis of expert radiologists. Current knowledge regarding the use of radiomics in diagnosing myxoid tumors is summarized in two prior studies [8,10]. These studies primarily focused on the radiomic features that showed efficacy in discriminating benign from malignant myxoid tumors. Martin-Carreras et al. in 2019 showed radiomic features from T1-weighted sequences discriminate better than signal intensity or tumor volume, with a key limitation that they did not use T2/STIR images in their model [10]. Chang et al. in 2022 reported T2 based geometry-based and wavelet-derived texture features were significantly different in benign and malignant tumors [8]. Both concluded that heterogeneity of T1 signal intensity was a useful discriminator. The six most important features in the ML model as determined by selection frequency by LASSO are shown in Fig 2. These consisted of one clinical feature (age), two intensity features (Range First Order_T1, and GrayLevelVariance_glrlm_T2), and three texture features (Clustershade_GLCM_T1, GrayLevelNonUniformityNormalized_glrlm_T2, and ClusterProminence GLCM_T1). Based on the magnitude of normalized value, the textural feature GrayLevelNonUniformityNormalized_glrlm_T2 had the largest association with malignancy. This factor meassured the variability of gray-level intensity values in a T2 weighted image, or the heterogeneity of the T2 signal. Though not the primary focus of the present study, future work will focus on validating the relevance of these features identified, potentially uncovering insights or confirming the importance of certain imaging characteristics.

More notable, the present study adds a robust performance comparison of the ML model to the consensus diagnosis by multiple expert musculoskeletal radiologists. In a smaller series of 39 patients, Chang et al. concluded that radiomics data had limited additive value to that of a single radiologist [8]. The overall similar diagnostic performance found between the ML model and the radiologists can generate one of two conclusions. Either that radiomic based interpretation of imaging is not useful because it was no better than radiologists, or that it is notable that it performed to the same level as three fellowship trained imaging experts. Though one may conclude it is a matter of perspective, our data indicates there may be clinical scenarios where ML augmentation of human interpretation of images may be useful. Though we found no overall difference between the radiomic-based ML model (78%) and radiologist (74%) accuracy, we performed additional subgroup analysis based on radiologist agreement and confidence. Another primary finding of this study was that radiologist confidence strongly correlated with their accuracy. Similar to prior work in benign fatty tumors, Nardo et al. reported when radiologists are confident, they are more accurate, as the percentage of incorrect MRI impressions correlated negatively with the radiologist confidence score [17]. Our data revealed the ML model may have unique utility to assist radiologists when they disagree, or when confidence is low. As these trends did not reach statistical significance, efforts are underway to increase the size of this imaging dataset to further investigate this.

Other potential application of radiomic based ML to assist in the diagnosis of soft tissue tumors is in the setting where musculoskeletal radiology or orthopaedic oncology expertise is not available. In that setting, an accessible, easy-to-use predictive model of similar accuracy to musculoskeletal radiologists could potentially augment the interpretation of local imaging specialists. It is well known that treatment of sarcomas at high-volume centers is associated with improved oncologic outcome, including overall survival and negative margin resection rate [18–20]. Of significant interest would be the potential to reduce misdiagnosis and improve referral to sarcoma centers for the diagnosis and treatment of these rare tumors. Further validation in larger and external (i.e., multi-institutional) cohorts is necessary prior to creation and evaluation of a tool that could be tested prospectively in a clinical setting.

This study has several limitations. Detecting differences in performance was limited by the small sample size of 90 cases, which is secondary to both the rare nature of these tumors and the stringent standard for image quality. Despite this, the study cohort is nearly twice as large as the second largest study of its kind [10]. Second, specific MRI protocols inevitably varied, due to the retrospective design, and inclusion of scans being performed at various outside institutions. Third, to maintain feasibility, more than one attending clinician performed the manual segmentation of images. For this reason, we employed one musculoskeletal radiologist to review all 90 segmentations to standardize acceptable quality prior to addition to the model. Fourth, in depth assessment of radiomic feature importance was outside the scope of our study question, which primarily focused on the comparison of the ML model to the radiologists. Fifth, given the limitation of sample size, we were unable to separately evaluate T2FS and STIR images, but grouped them together for the purpose of analysis. We recognize different distributions of these images could potentially reduce the performance of the ML model. Lastly, this data should not be overinterpreted, as it remains an internal validation of a single institution cohort for a specific type of soft tissue tumor. Multicenter collaboration to increase patient volume and provide external validity is warranted before this model should be tested in a clinical setting. Despite these limitations, the strength of this study includes the combined use of T2FS/STIR and contrast enhanced images, a three-dimensional approach to segmentation and analysis, and comparison to the consensus diagnosis by multiple radiologists, which add novelty in this research field.

## Conclusions

A radiomic based ML model predicted benign or malignant diagnosis in musculoskeletal myxoid soft tissue tumors similarly to consensus imaging interpretation by three musculoskeletal radiologists. Radiologist confidence in the diagnosis strongly correlated with their diagnostic accuracy. These data suggest that though radiomics and radiologists perform similarly overall, radiomics may provide novel diagnostic utility when radiologist confidence is low, or when radiologists disagree. The clinical significance and specific application of these findings needs further validation in a larger external cohort as we seek to create an easy-to-use tool that will assist clinicians in real-time with advanced image interpretation when the diagnosis of soft tissue tumors is in question.

## Supporting information

S1 Appendix (DOCX)
