# Supplementary material for: Distinguishing benign and malignant myxoid soft tissue tumors: Performance of radiomics vs. radiologists
Source: PLoS One. 2025 Jan 27;20(1):e0318072. doi: 10.1371/journal.pone.0318072 (PMC11771854; doi:10.1371/journal.pone.0318072)
Supplement: S1 Appendix — (DOCX) [file pone.0318072.s001.docx]

**Appendix**

1. A summary of the parameters tested and tuned during our experiments:

- **Random Forest**: The grid search was performed for the following parameters:
  - 'min_samples_leaf': [1, 2, 3]
  - 'n_estimators': [100, 300, 500]
  - 'max_features': ['auto', 'log2']
  - 'max_depth': [3, 5]
  - 'criterion': ['gini', 'entropy']
- **Logistic Regression**: We used penalty='l2' and solver='liblinear'. The grid search for 'C' was conducted over:
  - 'C': np.logspace(-2, 2, 100)
- **Multi-Layer Perceptron (MLP)**: Using solver='lbfgs', the grid search was conducted for:
  - 'learning_rate_init': [1e-04, 1e-03, 1e-02]
  - 'alpha': np.logspace(-3, 1, 5)
  - 'hidden_layer_sizes': [(64, 16), (64, 32, 16)]
- **XGBoost**: We set learning_rate=0.01, gamma=0, min_child_weight=1, subsample=0.8, colsample_bytree=0.8, reg_alpha=0.005, and random_state=3. The grid search was performed for:
  - 'n_estimators': [100, 300, 500]
  - 'max_depth': [3, 5]
- **Support Vector Classifier (SVC)**: With random_state=3, probability=True, and gamma='scale', the grid search for parameter 'C' was performed over:
  - 'C': np.logspace(-3, 2, 20)

1. **17 most frequently selected features (the normalized coefficients of these features are shown in figure 2):**

- T1-weighted images:

Cluster Shade (GLCM)

Range (First Order)

Cluster Prominence (GLCM)

Size-Zone Non-Uniformity (GLSZM)

Skewness (First Order)

Gray Level Variance (GLSZM)

Minimum (First Order)

Maximum (First Order)

- T2-weighted images:

Gray Level Variance (GLRLM)

Gray Level Non-Uniformity Normalized (GLRLM)

Inverse Variance (GLCM)

Inverse Difference (GLCM)

Informational Measure of Correlation 1 (GLCM)

Correlation (GLCM)

- Clinical Features:

Age
